# Antibiotic resistance in primary care in Austria - a systematic review of scientific and grey literature

**DOI:** 10.1186/1471-2334-11-330

**Published:** 2011-11-28

**Authors:** Kathryn Hoffmann, Gernot Wagner, Petra Apfalter, Manfred Maier

**Affiliations:** 1 Department of General Practice and Family Medicine, Center for Public Health, Medical University of Vienna, Waehringer Str. 13a/3rd floor, 1090 Vienna, Austria; 2 Institute for Hygiene, Microbiology and Tropical Medicine (IHMT), National Reference Centre for Nosocomial Infections and Antimicrobial Resistance, Elisabethinen Hospital Linz, Fadinger Str. 1, 4020 Linz, Austria

**Keywords:** Antibiotic resistance, primary health care, Austria, systematic literature review, grey literature

## Abstract

**Background:**

Antibiotic resistance is an increasing challenge for health care services worldwide. While up to 90% of antibiotics are being prescribed in the outpatient sector recommendations for the treatment of community-acquired infections are usually based on resistance findings from hospitalized patients. In context of the EU-project called "APRES - the appropriateness of prescribing antibiotic in primary health care in Europe with respect to antibiotic resistance" it was our aim to gain detailed information about the resistance data from Austria in both the scientific and the grey literature.

**Methods:**

A systematic review was performed including scientific and grey literature published between 2000 and 2010. Inclusion and exclusion criteria were defined and the review process followed published recommendations.

**Results:**

Seventeen scientific articles and 23 grey literature documents could be found. In contrast to the grey literature, the scientific publications describe only a small part of the resistance situation in the primary health care sector in Austria. Merely half of these publications contain data from the ambulatory sector exclusively but these data are older than ten years, are very heterogeneous concerning the observed time period, the number and origin of the isolates and the kind of bacteria analysed. The grey literature yields more comprehensive and up-to-date information of the content of interest. These sources are available in German only and are not easily accessible. The resistance situation described in the grey literature can be summarized as rather stable over the last two years. For *Escherichia coli *e.g. the highest antibiotic resistance rates can be seen with fluorochiniolones (19%) and trimethoprim/sulfamethoxazole (27%).

**Conclusion:**

Comprehensive and up-to-date antibiotic resistance data of different pathogens isolated from the community level in Austria are presented. They could be found mainly in the grey literature, only few are published in peer-reviewed journals. The grey literature, therefore, is a very valuable source of relevant information. It could be speculated that the situation of published literature is similar in other countries as well.

## Background

The increasing prevalence of antibiotic resistance (AR) is one of the major challenges for the healthcare systems worldwide. Antibiotic resistant infections are associated with a 1.3 to 2-fold increase in mortality compared to antibiotic susceptible infections [1]. If antibiotics become ineffective, infectious diseases will lead to an increase in morbidity and eventually premature mortality [2-4]. Moreover, AR imposes enormous health expenditure from higher treatment costs and longer hospital stays [5-10]. In addition, the development of new generations of antibiotic drugs is stalling [11]. Therefore, restrictive and appropriate use of antibiotics is even more needed to ensure the availability of effective treatment of bacterial infections. While up to 90% of antibiotics are being prescribed to patients in the outpatient sector existing information on the antibiotic resistance pattern is, with exceptions, based on samples from hospitalized patients [12]. Excessive use of antibiotics by humans, mainly due to antibiotic overtreatment of viral infections and in livestock breeding has led to a large output of resistant bacteria into the environment, where resistant bacteria and resistance genes can disseminate [13,14]. And indeed, the highest bacterial resistances rate was found where antibiotics are used most [12].

Two European initiatives provide valuable information on the topic "antibiotic resistance" in Austria: EARS-Net (formerly EARSS, European Antimicrobial Resistance Surveillance System) [15] and ESAC (European Surveillance of Antimicrobial Consumption) [16]. EARS-Net e.g. performs a continuous surveillance of antimicrobial susceptibility on the basis of laboratory analyses of invasive, blood-culture derived isolates from hospitalized patients. However, the antibiotic resistance pattern of the microbial flora of hospitalized patients differs from that seen in the community and, in addition, the resistance pattern of bacteria in routine primary health care is usually only tested after initial treatment failure [17]. Guidelines for prescribing antibiotics to patients at a community level should, therefore, be based on empirical and up to date evidence about antibiotic resistance of bacteria circulating in the community. Ideally, continuous surveillance of resistance patterns and antibiotic consumption in the outpatient setting should be carried out to detect changes.

In the year 2010 an EU-project called "APRES - the appropriateness of prescribing antibiotics in primary health care in Europe with respect to antibiotic resistance" started in nine European countries including Austria. One aim of this cross-sectional project is a systematic analysis of antibiotic resistance pattern of two key-bacteria at the community level. The analysis should be the basis for specific regional and national recommendations concerning the antibiotic prescribing behaviour of physicians in primary health care.

In the context of this EU-study we have undertaken a systematic literature review of all scientific papers and also of grey and non-English literature concerning the resistance pattern for the primary care sector in Austria in order to summarize existing facts and knowledge. It was our aim to assess strengths and weaknesses of the resistance situation at the community level described for Austria and to identify the sources and origin of the data published.

## Methods

### Study selection

A systematic literature review was performed of all available literature published between the 1^st ^of January 2000 and the 31^st ^January 2011. For the review process we followed the recommendations of the PRISMA (Preferred Reporting Items for Systematic Reviews and Meta- Analyses) statement [18] as it is described in the additional file 1. Three necessary inclusion criteria for the relevant literature were defined: First, the content has to deal with antibiotic resistance. Second, the resistance data have to be sampled in the ambulatory or community sector in humans and third, in Austria. We searched the scientific literature as well as the grey literature.

All types of indexed scientific literature were included. The bacteria included were *Streptococcus pyogenes*, *Streptococcus pneumoniae*, *Haemophilus influenzae*, *Moraxella catarrhalis*, *Klebsiella pneumoniae *for the respiratory tract, *Escherichia coli*, *Proteus mirabilis*, *Klebsiella *spp., *Staphylococcus saphrophyticus *for the urinary tract and *Staphylococcus aureus*. Literature containing resistance data from the hospital setting only and the literature not describing the origin either from hospital or primary care setting of the samples were excluded. The language of the literature included was English and German.

For the specification of the "grey" literature we used the definition of the Luxembourg Convention on Grey Literature: *"Grey literature is that which is produced on all levels of government, academics, business, and industry in print and electronic formats but which is not controlled by commercial publishers." *[19] Essentially, grey literature includes documents that have not been formally published in a peer-reviewed indexed format.

The literature search via electronic searches as well as the review process was carried out by two researchers (KH and GW) for the inclusion and exclusion criteria. Disagreement within the review process was resolved by discussion with the fourth author (MM).

### Search strategy

The literature search was performed during the period from April 1, 2010 until March 29, 2011.

For the scientific literature the databases and search engines PubMed, Medline and Embase were used. Search terms were the MeSH (Medical Subject Headings) terms "primary health care" OR "ambulatory care" AND "drug Resistance, Bacterial" AND "Austria" which were combined with the search terms "antibiotic resistance" OR "antimicrobial resistance", "primary care" OR "outpatient" OR "general practice" OR "community", in different combinations. According to the terms in English we used the corresponding German terms "Antibiotika", "Resistenzen", "Allgemeinmedizin", "niedergelassener Bereich" and "Österreich". Additionally, manual searches of the references of relevant articles including reviews were performed.

The search strategy for the grey literature was conducted via the search engines Google (http://www.google.at) and Google Scholar (http://scholar.google.at); in addition a systematic search on websites of institutions and organizations dealing with the sampling and determination of bacteria like regional laboratories for infectious diseases, reference centres or organizations that are responsible for public health like the Ministry of Health and linked facilities was performed. The search terms used were the same as for the scientific literature.

The exclusion process for the scientific literature was a three step process. The first step was the rejection of the duplicates, followed by the exclusion due to screening the title and abstract of papers identified; in the third step the papers were excluded by reading the full text of the papers, each of which was independently reviewed for eligibility. The exclusion process for the grey literature was performed as a three step process too by reading the "Google" title of the link and the short description first, followed by reading the full text of the relevant literature.

Finally, we allocated the literature that met all inclusion criteria into a "high quality" group (reports, government documents, recommendations of Austrian Societies, publications not published in an indexed journal) with respect to the method section described in this literature and a "low quality group" (interviews, official invitations, meeting notes) as recommended by Dobbins et al [20]. Only the "high quality" grey literature was included into this review.

### Data extraction

The outcome data extracted were: Numbers and characteristics of the included studies, bacteria types described, sampling location in Austria and general antibiotic resistance findings. Further, the sources of the data were documented.

## Results

**Figure 1:**
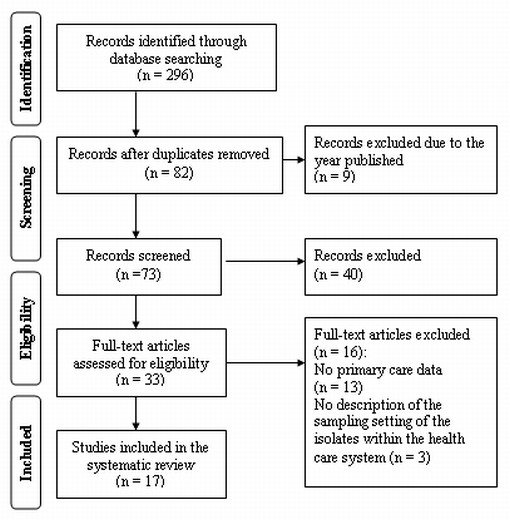
**PRISMA Flow Diagram**. The flowchart is showing the search strategies and the exclusion criteria used to locate the relevant scientific studies in this review.

After the rejection of the duplicate papers a total of 82 potential scientific papers were identified of which 9 were excluded on the basis of the year published and 40 were excluded after reading the abstract and title. Further 16 papers were excluded after reading the full text. Most of the papers were excluded because the reported incidence of resistance data was exclusively from the hospital sector in Austria (EARS-Net) or other countries than Austria. Seventeen papers were included into the final review. Figure 1 shows the "PRISMA Flow Diagram" for the scientific literature results [18]. The grey literature search strategy yielded e.g. 3,840 potential relevant links on March, 29 2011 by using the combination of the search terms "Antibiotikaresistenzen" AND "Österreich" AND "niedergelassener Bereich" in Google.at. After the three step review procedure 23 high quality publications remained of which 21 are resistance reports (19 regional reports from different years published between 2002 and 2011 and two national reports of the years 2008 and 2009). Most of the excluded literature enunciated treatment recommendations of infectious diseases for the ambulatory sector by referencing to data from the hospital sector.

Table 1 summarizes the basic characteristics and selected resistance findings of the final 17 scientific papers. Nine [21-29] out of the 17 scientific papers describe the resistance pattern of certain bacteria for the ambulatory sector only by referencing to four data sources, the Alexander-Project [22,27], the PROTEKT (Prospective Resistant Organism Tracking and Epidemiology for the Ketolide Telithromycin) surveillance study [21,23,25], the ECO-SENS (International Survey of the Antimicrobial Susceptibility of Urinary Pathogens) project [24,26,29] and the ARESC (Antimicrobial Resistance Epidemiological Survey on Cystitis) study [28]. Eight of these nine publications describe the resistance situation in Austria before the year 2001. The reported resistance patterns of the analysed bacteria in the other publications are based on isolates from both the hospital and the outpatient sector in different proportions.

**Table 1:** Basic characteristics and general resistance findings of the final 17 scientific papers (in alphabetical order)

Author	Sampling setting and report of results	Sampling location	Years of resistance testing	Total no. of isolates (Austria)	Bacteria	Antibiotics	General Resistance findings
Auer et al 2010 [30]	Hospital (2%) and primary health care (98%) - results reported together	Salzburg, Upper Austria, Styria (Austria)	2004-2008	100	*ESBL-producing E. coli*	FOF, MEL, ETP, NIT, SXT, GEN, CIP	3% FOF, 6% NIT, 15% MEL, 0% ETP, 22% GEN, 73% SXT, 78% CIP^a^

Badura et al 2007 [37]	Hospital and primary health care- results reported together	Southeast Austria	1997-2006	690,967 collectively	*E. coli, S. aureus, Klebsiella *spp.	Various for each bacteria	The data show insignificant changes in prevalence of *MRSA *and vancomycin-resistant enterococci in southeast Austria during the past decade (1997-2006) but an alarming increase *of ESBL-producing E. coli *isolates in recent years.

Buxbaum et al 2003 [32]	Hospital and primary health care- results reported together	Austria	2001-2002	542	*S. pneumoniae*,	PEN, TEL, ERY, CLR,	2.2% PEN, 0% TEL, 8.5% ERY, 10.3% CLR, 7% AZM
				
				223	*S. pyogenes*,	same	0% PEN, 0% TEL, 8% ERY, 6.7% CLR, 8.1% AZM
				
				183	*S. aureus*,	same	73.2% PEN, 2.2 TEL, 17% ERY, 16.4% CLR, 16.4% AZM
				
				67	*H. influenzae*	AMP instead of PEN	1.5% AMP, 0% TEL, ERY, CLR, AZM

Canton et al 2002 [21]	Primary health care	25 countries worldwide incl. Austria	1999-2000	25	*S.pyogenes*,	ERY, LVX, PEN, TEL	No special results for Austria. But compared to the other countries Austria had one of the lowest resistance rates.
				
				20	*S.aureus*	TEL	0% TEL

Cizman 2003 [22]	Primary health care	21 countries in Europe incl. Austria	1997-1999 1997-2000	n/a	*H. influenzae*,	PEN	The antibiotic resistance rates were set in correlation with the mean national outpatient consumption. Compared to the other countries Austria had a low total outpatient consumption of 13.80 DDD/1000 inhabitants/day in 1997 and a penicillin resistance rate of *S. pneumoniae *of 12.4%. With the consumption of macrolides Austria was ranked in the middle field with a resistance rate for *S. pneumoniae *of 11.4%.
						
					*S. pneumoniae*,	PEN, ERY	
						
					*S. pyogenes*	ERY	

Felmingham et al 2002 [23]	Primary health care	25 countries worldwide incl. Austria	1999-2000	57	*S. pneumoniae*	PEN, ERY	5.3% PEN, 12.3% ERY Compared to the other countries Austria had one of the lowest resistance rates.

Graninger 2003 [24]	Primary health care	16 European countries incl. Austria and Canada	1999-2000	n/a	*E. coli*	n/a	The publication highlights the effectiveness of MEL compared to other antibiotics

Hoban et al 2002 [25]	Primary health care	25 countries worldwide incl. Austria	1999-2000	40 19	*H.influenzae* *M. catarrhalis*	n/a	2.5% ß-lactamase + 89.5% ß-lactamase +

Hönigl et al 2010 [33]	Hospital (63%) and primary health care (37%)- results reported together	Southeast Austria	1997-2008	1997: (n = 113) 2008: (n = 218)	*S. pneumoniae*	PEN, ERY, CLI, TET, SXT, quinolones	1997: 3.5% ERY, 1.8% CLI, 1.8% TET, 7.1% SXT, 0.9% QUIN 2008: 14.7% ERY, 10.6% CLI, 11% TET, 9.2% SXT, 0.5% quinolones

Kahlmeter 2003 [26]	Primary health care	17 countries in Europe incl. Austria	1999-2000	126	*E. coli*	AMP, AMC, MEC, CFR, TMP, SUL, SXT, NAL, CIP, NIT, FOF, GEN	Compared to the other 16 European countries Austria had one of the lowest resistance rates for *E. coli*: 17.5% AMP, 2.4% AMC, 1.6% MEC, 0.8% CFR, 9.5% TMP, 25.4% SUL, 9.5% SXT, 2.4% NAL, 0% CIP, 0.8% NIT, 0% FOF, 0.8% GEN

Kahlmeter et al 2003 [29]	Primary health care	17 countries in Europe including Austria	1999-2000	126	*E. coli*	AMP, AMC, MEC, CFR, TMP, SUL, SXT, NAL, CIP, NIT, FOF, GEN	17.5% AMP, 2.4% AMC, 1.6 MEC, 0.8% CFR, 9.5% TMP, 25.4 SUL, 9.5% SXT, 2.4% NAL, 0% CIP, 0.8% NIT, 0% FOF, 0.8 GEN

Krziwanek et al 2008 [35]	Hospital and primary health care- results reported together	Austria	1996-2006	1,439	*MRSA*	n/a	In Carinthia, 73% of all *MRSA *belonged to ST228. In the Austrian region "Salzkammergut", the proportion of ST5 increased from 26% in 2004 to 89% in 2006. In eastern Upper Austria and western Lower Austria, the ST8 Austrian clone was predominant.

Krziwanek et al 2009 [36]	Hospital and primary health care- results reported together	Upper Austria	2006-2008	1,098	*MRSA*	n/a	Out of the 1,098 *MRSA *samples from humans, 21 were *MRSA *type ST398 that is usually associated with animals. Most of these 21 patients were farmers (n = 16). Increasing prevalence from 1.3% in 2006 to 2.5% in 2008 shows emergence of *MRSA *ST398 in humans in Austria.

Prelog et al 2008 [31]	Hospital and primary health care- results reported together	Western Austria	2006	2,042	*E. coli*	n/a	20 out of the 2,042 *E. coli *isolates demonstrated alleles encoding CTX-M enzymes belonging to phylogentic group 1.

Schito et al 2000 [27]	Primary health care	14 countries in Europe incl. Austria	1992-1998	185	*S. pneumoniae*	PEN, ERY	4.8% PEN, 11.4%
				
				153	*H. influenzae *	DOX, SXT, CIP	1.3% DOX, 13.7% SXT, 0.0% CIP
				
				n/a	*M. catarrhalis*	n/a	

Schito et al 2002 [34]	Hospital and primary health care- results reported together	Italy, Spain, Austria	1999-2000	3,593 collectively in all three countries	*S. pneumoniae*, *M. catherrralis*, *H. influenzae* *K. pneumoniae* *S. pyogenes*, *S. aureus*	AMP, AMC, CEC, CXM, CFM, CTB, CPD, AZM, CLR for all bacteria	The results show a substantial prevalence of macrolide resistance of the bacteria analysed in Italy, Spain and Austria.

Schito et al 2009 [28]	Primary health care	9 European countries including Austria and in addition Brazil	2003-2006	3,018 collectively in all nine countries	*E. coli, K. pneumoniae, P. mirabilis, S. saphrophyticus*	AMP, AMC, MEC, CFX, NAL, CIP, SXT, NIT, FOF	Mean resistance rates for *E.coli *between 2003 and 2006 for Austria were e.g.: 48.3% AMP 8.1% NAL and 29.0% SXT. Compared to the other countries Austria with 48.3% resistance against AMP had one of the highest resistance rates; against the other antibiotics one of the lowest rates.

Abbr.: FOF, fosfomycin; MEL, pivmecillinam; ETP, ertapenem; NIT, nitrofurantoin; GEN, gentamicin; SXT, trimethoprim-sulfamethoxazole; CIP, ciprofloxacin; PEN, penicillin; ERY, erythromycin; CLI, clindamycin; TET, tetracycline; AMP, ampicillin; AMC, co-amoxiclav; MEC, mecillinam; CFR, cefadroxil; TMP, trimethoprim; SUL, sulfamethoxazole; NAL, nalidixic acid; DOX, doxycyclin; CXM, cefuroxime; CEC, cefaclor; CFM, cefixime; CTB, ceftibuten; CPD, cefpodoxime; AZM, azithromycin; CLR, clarithromycin; LVX, levofloxacin

n/a: Data not described in the publication

^a^: Resistance data include intermediate susceptible isolates

Six articles show the resistance pattern of *E. coli*, *ESBL-producing E. coli *and other bacteria related to community-acquired uncomplicated urinary tract infections [24,26,28-31], eight the resistance pattern of the bacteria included for community-acquired respiratory tract infections [21-23,25,27,32-34], further two are dealing with the *MRSA *situation in Austria [35,36] and one article is a "letter to the editor" with an overview of multiple bacteria [37]. No overall conclusion of the current resistance situation in the ambulatory sector in Austria can be gathered out of these different studies due to the differences in sampling settings, inclusion and exclusion criteria of the study population, time periods, bacteria analysed and the methodology used. Moreover, the determination of the resistance rates in Austria was conducted using the CLSI (Clinical and Laboratory Standards Institute) standard. Within this standard there has been a change in the antimicrobial MIC breakpoint in 2008 which means that most data before and after 2008 are not comparable [38].

Tables 2 and 3 summarize the basic characteristics and selected resistance findings of the 23 high quality grey literature documents of which only the isolates from the primary health care sector included into the reports are described. The resistance findings included are contained in several reports and their updates since the year 2008 separately for the ambulatory and the hospital sector. The key-bacteria analysed for the urinary and respiratory tract are the same among the reports. The Austrian resistance reports (AURES) of the years 2008 and 2009 e.g. include the chapter "Resistance report for selected non-invasive microbial pathogens" which summarize the data from the ambulatory sector of several large microbiology laboratories from all over Austria; a change in the resistance pattern of the bacteria included can be observed in certain regions over several years (table 3). While the change in the CLSI standard in 2008 has to be considered for these resistance reports as well the overall resistance situation in the primary care sector in Austria for e.g. *E. coli *is summarized as following: *"The percentage of the ESBL-producing E. coli is with 6% stable over the last two years in the primary care sector. The highest antibiotic resistance rates for E. coli and ESBL-producing E. coli can be seen with fluorochinolones (19%/85%) and trimethoprim/sulfamethoxazole (27%/82%)."*[39]

**Table 2:** Basic characteristics of the 23 high quality grey literature documents

Editor	Title	Sampling setting and report of the results	Sampling location	Tested bacteria	Years of sampling and resistance testing
Bundesministerium f. Gesundheit Österreich	Österreichischer Resistenzbericht AURES 2009 [39]	Hospitals and primary health care sector - results reported separately	Austria	*S. pyogenes, S. Pneumoniae* *H.influenzae* *E. coli, P. mirabilis*, *S. aureus*	One year before the publication
					
	Österreichischer Resistenzbericht AURES 2008 [61]				

Abt. f. Mikrobiologie, Med.-chem. Labor Dr. Mustafa, Labor Dr. Richter OG	Resistenzreport 2009 Zusammenfassung der lokalen Resistenzdaten [62]	The majority of isolates was collected in the primary health care sector but some isolates were collected in hospitals and residencies as well - results reported partly separate since 2009	Salzburg, Upper Austria, Upper Styria (Austria)	*S. pyogenes, S. pneumoniae* *H. influenzae* *E. coli, P. mirabilis*, *S. aureus *	One year before the publication
					
	Resistenzreport 2008 Zusammenfassung der lokalen Resistenzdaten [63]				
					
	Resistenzbericht 2007 [64]				
					
	Resistenzbericht 2006 [65]				
					
	Resistenzbericht 2005 [66]		Salzburg (Austria)		
					
	Resistenzbericht 2004 [67]				
					
	Resistenzbericht 2003 [68]				
					
	Resistenzbericht 2002 [69]				

Institut f. Hygiene, Mikrobiologie und Umweltmed. Med. Univ. Graz, Labor für Med. Bakteriologie und Mykologie	Resistenzbericht 2010 [70]	Isolates from primary health care (50%) and from secondary and tertiary care sector (50%) - results reported partly separate	Styria (Austria)	*S. pyogenes, S. pneumoniae* *H. influenzae* *E. coli, P. mirabilis*, *S. aureus*	One year before the publication
					
	Resistenzbericht 2009 [71]				
					
	Resistenzbericht 2008 [72]				

Bakterielles Labor des LKH Leoben	Resistenzbericht 2009 aus dem Einsendegut des Bakt. Labors im LKH Leoben [73]	Hospitals (90%) and primary health care (10%) - results reported partly separate since 2007	Upper Styria (Austria)	*S. pyogenes, S. pneumoniae* *H. influenzae* *E. coli, P. mirabilis*, *S. aureus*	One year before the publication
					
	Resistenzbericht 2007 aus dem Einsendegut des Bakt. Labors im LKH Leoben [74]				
					
	Resistenzbericht 2006 aus dem Einsendegut des Bakt. Labors im LKH Leoben				
					
	Resistenzbericht 2005 aus dem Einsendegut des Bakt. Labors im LKH Leoben [75]				
					
	Resistenzbericht 2004 aus dem Einsendegut des Bakt. Labors im LKH Leoben [76]				
					
	Resistenzbericht 2003 aus dem Einsendegut des Bakt. Labors im LKH Leoben [77]				

Sekt. Hygiene u. Med. Mikrobiologie u. Univ. Klinik für Innere Med. I, Klin. Infektiologie u. Immunologie Med. Univ. Innsbruck	Resistenzbericht 2009. Resistenzverhalten von Bakterien und Pilzen gegen Antibiotika und Antimykotika [78]	Primary, secondary and tertiary health care - results reported separately	Tyrol (Austria)	*S. pyogenes, S. pneumoniae* *H.influenzae* *E. coli, P. mirabilis*, *S. aureus *	One year before the publication
					
	Resistenzbericht 2008. Resistenzverhalten von Bakterien und Pilzen gegen Antibiotika und Antimykotika [79]				

Hell 2010 [80]	ESBL-producing E.coli in uncomplicated UIT - regional and Austria-wide update and evaluation of treatment options	Primary health care	Austria	*ESBL-producing E. coli*	2004-2008

Grisold 2011 [81]	Das Antibiogramm: Indikation - Interpretation - Qualität	Hospitals and primary health care sector - results reported separately	Styria (Austria)	*ESBL-producing E. coli*	n/a

**Table 3:** Selected resistance findings from the ambulatory sector only of the most up-to-date grey literature included

Location	Pathogen	Tested isolates		Antibiotics	Resistance in %		Additional information	
**2009 and 2008 AURES **[39,61]**Bundesministerium f. Gesundheit Österreich**								

		**2008**	**2009**		**2008**	**2009**	**2008**	**2009**

Respiratory tract	*S. pyogenes*	977	1,440	PEN	0	0		
				
		1,436	1,433	macrolides	3.6	3.3		
				
		1,438	1,356	fluoroquinolones	0.7	0.3		
			
	*S. pneumoniae*	510	454	PEN	0.8	1.8		
				
		510	454	macrolides	14.5	13.4		
				
		506	455	fluoroquinolones	0.8	0.2		
			
	*H. influenzae*	1,244	1,255	AMP or AMX	9.4	9.6		
				
		1,244	1,255	AMC	0	0.1		
				
		1,244	1,234	fluoroquinolones	0.2	0		

All locations	*S. aureus*	2,395	3,970	OXA	2.4	2.1	MRSA 2.4%	MRSA 2.1%
				
		2,994	3,746	macrolides	14.5	14.9		
				
		3,045	3,757	CLI	6.9	9.7		
				
		3,277	3,547	SXT	0,8	0.6		
				
		1,633	3,886	fluoroquinolones	3.8	3.6		

Urinary tract	*E coli*	8,992	11,218	AMP or AMX	39.8	43.9	ESBL 6.0%	ESBL 6.4%
				
		8,985	11,219	AMC	5.8	9.2		
				
		9,088	11,107	cephalosporin 1^st^	8.5	9.7		
				
		8,992	11,225	SXT	24.6	27.5		
				
		8,992	11,241	fluoroquinolones	15.7	18.8		
				
		8,789	10,738	NIT	2.2	2.7		
				
		4,361	4,893	MEL	12.2	6.8		
				
		5,489	7,799	FOF	1.5	2.3		
			
	*P.mirabilis*	n/a	n/a					

**Location**	**Pathogen**	**Tested isolates**		**Antibiotics**		**Resistance in %**	**Additional information**	

**2010 Medical University Graz, Department for Hygiene and Microbiology **[70]								

Respiratory tract	*S. pyogenes*	n/a						
			
	*S. pneumoniae*	n/a						
			
	*H. influenzae*	n/a						
	
	*S. aureus*	444		OXA	2.5		MRSA 2.5%	
				
		441		SXT	0			
				
		434		CIP	3			
				
		444		ERY	14.9			
				
		444		CLI	13.7			

Urinary tract	*E. coli*	3,907		AMX	61.4		ESBL 7.4%	
				
		3,907		AMC	37.5			
				
		3,902		CFX	6.3			
				
		3,907		TMP	27.6			
				
		3,907		SXT	27.2			
				
		3,881		FOF	0.8			
				
		3,907		CIP	18.5			
				
		3,905		NIT	0.4			

**2009 Laboratory Dr Richter and Dr Mustafa, Section for Microbiology, Salzburg **[62]								

Respiratory tract	*S. pyogenes*	n/a						
			
	*S. pneumoniae*	n/a						
			
	*H. influenzae*	n/a						

Skin and soft tissue	*S. aureus*	938		OXA	3		MRSA 3%	
				
		938		AMC	3			
				
		938		CFX	3			
				
		938		ERY	13			
				
		938		CLIN	3			
				
		938		SXT	1			
				
		938		MXF	3			

Urinary tract	*E. coli*	2,506		AMP	40		ESBL 4%	
				
		2,506		AMC	6			
				
		2,506		CFX	6			
				
		2,506		SXT	26			
				
		2,499		NIT	1			
				
		2,506		CIP	16			
				
		2,504		FOF	1			
			
	*P. mirabilis*	n/a						

**2009 Medical University Innsbruck, Section for Hygiene and Medical Microbiology **[78]								

Respiratory tract	*S. pyogenes*	187		PEN	0			
				
		187		AZM	5.8			
				
		187		MXF	2.1			
			
	*S. pneumoniae*	115		PEN	0			
				
		115		AZM	12.1			
				
		115		MXF	0.87			
			
	*H. influenzae*	76		AMP	25			
				
		76		AMC	0			
				
		76		MXF	0			

All locations	*S. aureus*	676		FOX	1.48		MRSA 6%	
				
		610		AZM	20.9			
				
		610		CLIN	18			
				
		674		SXT	1.03			
				
		609		MXF	1.97			

Urinary tract	*E. coli*	3,112		AMP	58		ESBL 9%	
				
		3,104		AMC	20			
				
		3,101		CFZ	17			
				
		3,112		SXT	33			
				
		3,112		CIP	26			
				
		3,097		MEC	7			
				
		3,111		NIT	7			
**2009 County Hospital Leoben, Bacteriological Laboratory **[73]								

Respiratory tract	*S. pyogenes*	n/a						
			
	*S. pneumoniae*	n/a						
			
	*H. influenzae*	n/a						
	
	*S. aureus*	141		AMC	1.4			
				
		141		OXA	1.4			
				
		141		CFZ	1.4			
				
		140		CLIN	11.4			
				
		140		ERY	12.1			
				
		140		CIP	0.7			

Urinary tract	*E. coli*	310		AMP	41			
				
		310		AMC	10			
				
		310		CFZ	10.0			
				
		309		TMP	29.5			
				
		310		CIP	20,0			
				
		309		NIT	1.9			
				
		27		MEC	11.1			
	
	*P. mirabilis*	28		AMC	10.7			
				
		28		CFZ	17.9			
				
		28		TMP	53.6			
				
		28		CIP	17.9			

**Hell **[80]								

Urinary tract	*E. coli* *ESBL-producing *	100		MEC	11			
		100		FOF	3			
				
		100		NIT	1			
				
		100		SXT	73			
				
		100		CIP	78			

Abbr.: FOF, fosfomycin; MEL, pivmecillinam; NIT, nitrofurantoin; SXT, trimethoprim-sulfamethoxazole; CIP, ciprofloxacin; PEN, penicillin; ERY, erythromycin; CLI, clindamycin; AMP, ampicillin; AMX, amoxicillin; AMC, amoxicillin/clavulanate; MEC, mecillinam; TMP, trimethoprim; SUL, sulfamethoxazole; AZM, azithromycin; CFZ, cefazolin; FOX, cefoxitin; OXA, oxacillin; MXF, moxifloxacin

n/a: Resistance data of isolates from primary care alone not available

All resistance reports included are published in German only and on special websites.

## Discussion

This review provides the most comprehensive and up to date information on the pattern of AR at the community level in Austria. This has been achieved by a thorough search of both the scientific and grey literature.

Our analysis shows that the seventeen scientific publications included describe only a small part of the resistance situation in the primary health care sector in Austria (table 1). Half of these publications contain data from the ambulatory sector only but are older than ten years. Moreover, the included scientific literature is very heterogeneous concerning the observed time period, the number and origin of the isolates and the kind of bacteria analysed.

In contrast, the grey literature yields more substantial information on the content of interest (tables 2 and 3). Mainly the "resistance reports" (Resistenzberichte) contain comprehensive and up-to-date resistance data from the ambulatory level. Since 2008, the AURES report in particular is the only source which provides comprehensive, structured and nationwide data on a yearly basis from isolates obtained exclusively at the primary care sector. The resistance situation described can be summarized as rather stable over the last two years. A comparison of the resistance situations can be drawn on a regional and on a national level (table 3). This literature is at the moment the best available source of ambulatory resistance data; however, the data are not covering all regions of Austria. Further, the reports are available in German only, are accessible on certain specific websites only and are not published in indexed journals. Therefore, it is nearly impossible for someone who cannot speak German or is not familiar with the website address to find a comprehensive source of information about the current resistance situation in the primary health care sector in Austria. Moreover, the methodology for the determination of resistance differs between Austrian counties and European countries which, hopefully, will improve due to the EUCAST (European Committee on Antimicrobial Susceptibility Testing) efforts to harmonize the MIC breakpoints for antimicrobial susceptibility testing of bacteria in Europe [40]. This could be an obstacle for comparative studies which are based on systematic literature searches from different countries or for finding adequate sources to describe the status quo in Austria. Unfortunately, the results from the promising international studies (Alexander project and PROTEKT study)[41] of the years 1999 and 2000 that dealt with the resistance pattern of bacteria responsible for community-acquired infections of the respiratory tract have not been translated into regular national or international surveillance systems.

In contrast, comprehensive and current antibiotic resistance data from the hospital sector or outpatient antibiotic consumption data in Austria are easy to find in the scientific literature due to the longstanding partnership of Austria with the EARS-Net and ESAC network. These standardized data were collected nationwide and published regularly [42,43]. This could be the reason that, at the moment, all recommendations available for the treatment of community-acquired infectious diseases for the primary health care sector still are based on resistance data derived from the hospital sector [44]. Examples are the brochure of the Austrian Antibiotic Stewardship Group (ABS) for the ambulatory sector or the latest expert consensus of the Austrian Society of Infectious Diseases concerning Antibiotic treatment in primary health care [45,46]. It should be mentioned that first steps towards a resistance-register for non-invasive isolates from the primary health care sector in Austria are already under way. The collection and reporting of non-invasive isolates and their resistances in the regional and the national resistance reports in a structured and systematic way could be the starting point. Also the ABS group is advancing this issue [47,48].

The strengths of this review are its focus on the outpatient setting in Austria and its comprehensiveness by the inclusion of both the scientific and the grey literature. In fact, for our purpose the data contained in the grey literature prove very valuable in achieving our aim of comprehensiveness and reduces publication and selection bias. It could be speculated that the situation of published literature is similar in other countries as well. However, recent studies have examined the impact of the inclusion of the grey literature in systematic reviews to describe the status quo of a situation in more detail [49-51]. Especially, since a Cochrane update was published in 2004 to highlight the importance of widespread literature search strategies for public health interventions including the grey literature for systematic reviews [52] the number of reviews which include grey literature is growing constantly in many health related sectors [53-60].

The limitations of this literature review are the reliance on previously published research results. Even more difficult is the reliance on the grey literature found due to the non hierarchic search results with the search engine Google. Moreover, in this review we compared the scientific and the grey literature and draw conclusions on their comprehensiveness and on the public health relevance of their content. Since the scientific papers were independently peer-reviewed and the grey literature was published by organizations without that review process this may affect the methodological quality and therefore, the scientific level of evidence. In addition, it is not possible to directly compare the resistance data described in the scientific and grey literature: in the scientific literature the resistance data are mainly analysed for specific diseases like e.g. uncomplicated urinary tract infections in a defined group of patients, in the grey literature the resistance data reported are the result of all isolates analysed for one specific bacterium regardless of a given disease.

## Conclusion

In this review, comprehensive and up-to-date antibiotic resistance data of different pathogens, isolated from the primary care level in Austria, are presented. They could be found mainly in the grey literature, only few are published in peer-reviewed journals. The grey literature, therefore, is a very valuable source of relevant information and might reveal possibilities for further research.

Based on these findings we recommend collecting and publishing also the non-invasive resistance findings on a regular basis in indexed journals like it is done in the EARS-Net and ESAC network.

## Competing interests

The authors declare that they have no competing interests.

## Authors' contributions

KH and GW performed the literature search; KH performed the extraction of the data and drafted the initial manuscript, GW helped with the extraction of the grey literature data; MM conceptualized the study and helped with data interpretation; PA and MM critically revised the manuscript for important content. All authors read and approved the final manuscript.

## Pre-publication history

The pre-publication history for this paper can be accessed here:

http://www.biomedcentral.com/1471-2334/11/330/prepub

## Supplementary Material

Additional file 1 **PRISMA 2009 checklist**. Checklist including relevant page numbers for identifying various components of the review. Click here for file
